# Structural and antitrypanosomal data of different carbasones of piperitone

**DOI:** 10.1016/j.dib.2016.11.044

**Published:** 2016-11-18

**Authors:** Amoussatou Sakirigui, Fernand Gbaguidi, Urbain C. Kasséhin, Jacques Poupaert, Georges C. Accrombessi, Simeon O. Kotchoni

**Affiliations:** a University of Abomey-Calavi (UAC), Faculty of Sciences and Technics (FAST), Department of Chemistry, Laboratory of Physic and Synthesis Organic Chemistry (LaCOPS), 01 PB: 4521, Cotonou, Benin; b Laboratoire de Pharmacognosie, Centre Béninois de Recherche Scientifique et Technique, 01 PB 06 Oganla, Porto-Novo, Benin; c Laboratoire de Chimie Pharmaceutique Organique, Faculté des Sciences de la Santé, Université d׳Abomey-Calavi, Campus du Champ de Foire, 01 BP 188, Cotonou, Bénin; d Université catholique de Louvain (UCL), Louvain Drug Research Institute (LDRI), B1 7203 Av. E. Mounier 72, B-1200 Bruxelles, Belgium; e Department of Biology, Rutgers University, 315 Penn St., Camden, NJ 08102, USA; f Center for Computational and Integrative Biology, 315 Penn St., Camden, NJ 08102, USA

**Keywords:** Hemi-synthesized, Piperitone carbazones, Essential oil, *Cymbopogon shoenantus*, *Trypanosoma brucei brucei*, *Artemia salina*

## Abstract

This article reports data on four carbazones of piperitone: semicarbazone **1**, thiosemicarbazone **2**, 4-phenyl semicarbazone **3** and 4-phenyl thiosemicarbazone **4** prepared directly *in situ* from essential oil of *Cymbopogon schoenantus,* whose GC-FID and GC–MS analysis revealed piperitone as major component (68.20%). The structures of hemi-synthesized compounds were confirmed by high throughput IR, MS, ^1^H and ^13^C NMR based spectrometric analysis. Their antiparasitic activities were evaluated *in vitro* on *Trypanosoma brucei brucei* (*Tbb*). The compound **3** (IC_50_=8.63±0.81 µM) and **4** (IC_50_=10.90±2.52 µM) exhibited antitrypanosomal activity, **2** had a moderate activity (IC_50_=74.58±4.44 µM) but **1** was void of significant activity (IC_50_=478.47 µM). The *in vitro* tests showed that all compounds were less cytotoxic against the human non cancer fibroblast cell line (WI38) (IC_50_>80 µM) while only **2** (IC_50_=21.16±1.37 μM) and **4** (IC_50_=32.22±1.66 µM) were cytotoxic against the Chinese Hamster Ovary (CHO) cells and toxic on *Artemia salina* (Leach) larvae. Piperitone 4-phenyl semicarbazone **3**, the best antitrypanosomal compound, showed also a selectivity index (SI) higher than 7 on the larvae and the tested cells and therefore might be further studied as antitrypanosomal agent. Also, all compounds except **3** showed selectivity between the two tested cell lines (SI>2). This data reveals for the first time the antitrypinosomal properties of thiosemicarbazones, their cytotoxicity on mammalian cells as well as their activities against *Tbb* and *A. salina* Leach.

**Specifications Table**

**Table:** 

Subject area	*Chemistry, Biology, Phytochemistry, Analytical Chemistry, Medicinal Biology*
More specific subject area	*Pharmacognosy*
Type of data	*Tables, text file, figures*
How data was acquired	*FT-IR (Perkin-Elmer Frontier 286*^*™*^*), GC–MS (Thermo-Quest), NMR (Bruker), in vitro bioassays*
Data format	*Analyzed*
Experimental factors	*Structural elucidation and antitrypanosomal activities of novel compounds derived from medicinal plants*
Experimental features	*Thiosemicarbazones were sysnthesized from Cymbopogon schoenatus essential oil and fully characterized using GC–MS, FT-IR, and NMR and their* antiparasitic activities evaluated *in vitro* on *Trypanosoma brucei brucei* (*Tbb*)
Data source location	*Cotonou, Benin*
Data accessibility	*The data is available with this article*

**Value of the data**

• 4-phenyl semicarbazone (**3)** and 4-phenyl thiosemicarbazone (**4)** can be used as asantitrypanosomal drugs against sleeping sickness.
• 4-phenyl semicarbazone (**3)** can be used with no cytotoxicity effect.
• *Data shows that Cymbopogon schoenatus* essential Oil can be used as an antiparasitic agent.

## 1 Data

The data of this study provides the chemical composition characteristics (Supplementary data), the antitrypanosomal activities and the cytotoxicity levels of novel *in situ* hemisynthesis of thiosemicarbazone derivatives from *Cymbopogon schoenatus* essential oil (Table 1, Table 2).

## 2 Experimental design, materials and methods

### 2.1 Analysis of the essential oil by GC-FID and GC–MS

The GC-FID analysis was carried out on a FOCUS GC (Thermo Finigan; Milan, Italy) using the following operating conditions: HP 5MS column (30 m×0.25 mm, film thickness: 0.25 μm) (J&W Scientific Column of Agilent Technologies, USA); injection mode: splitless; injection volume: 1 µL (TBME solution); flow of split: 10 ml/min; splitless time: 0.80 min; injector temperature: 260 °C; oven temperature was programmed as following: 50 °C–250 °C at 6 °C/min and held at 250 °C for 5 min; the carrier gas was helium with a constant flow of 1.2 mL/min; FID detector temperature was 260 °C. The data were recorded and treated with the ChromCard software. The quantification was completed by the calculation of the areas under curve of the peaks (GC-FID, normalization process) and the identification of compounds by comparison of the retention indices (RI) with the references. The GC–MS analysis were carried out using a TRACE GC 2000 series (Thermo-Quest, Rodano, Italy), equipped with an autosampler AS2000 Thermo-Quest operating in the electronic impact mode at 70 eV. HP 5MS column (30 m×0.25 mm, film thickness: 0.25 μm). The coupling temperature of the GC was 260 °C and the temperature of the source of the electrons was 260 °C. The data were analyzed with the Xcalibur 1.1 software (ThermoQuest). The mass spectra of the peaks were analyzed and compared with references, literature and the NIST/EPA/NIH database. The individual components of the volatile oils were identified by comparison of their relative retention times with those of authentic standard references, computer matching against commercial library and custom proprietary library mass spectra made from pure substances and components of known oils. Mass spectrometry literature data were also used for the identification. Quantification (expressed as percentages) was carried after normalization using peak areas obtained by FID.

### 2.2 Hemi-synthesized compounds identification

**Fig. 1:**
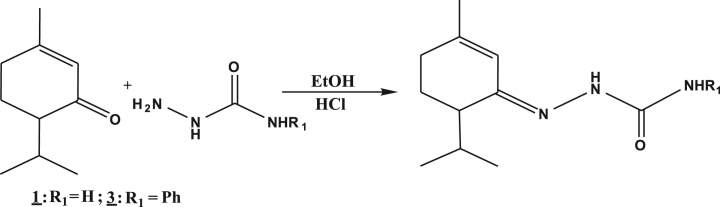
Hemi-synthetic routes of semicarbazones.

**Fig. 2:**
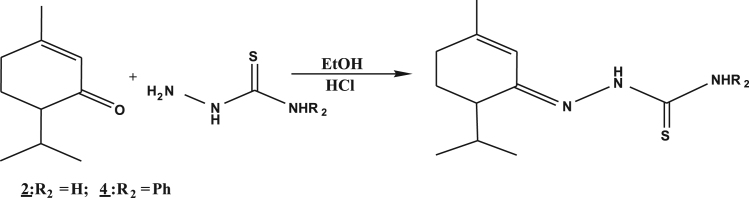
Hemi-synthetic routes of thiosemicarbazones.

The melting points were taken on a fusionometer type *electrothermal 1A 9000*. The IR spectra were recorded on a Perkin-Elmer FTIR 286. The frequencies of absorption bands were expressed in cm^−1^. The NMR spectra were registered on a Bruker 500 in chloroform-d6 (CDCl_3_) or dimethylsulfoxide-d6 (DMSO-d6) which frequencies for ^1^H and ^13^C were 400 MHz and 100 MHz respectively. Chemical shifts were given in parts per million (ppm) relative to tetra-methyl silane (TMS) as an internal reference. Multiplicity was designated as singlet (s), triplet (t), doublet (d) and multiplet (m). MS spectrometric data of compounds were reported in APCI mode. The semicarbazones and thiosemicarbazones were synthesized by the following methods: Piperitone semicarbazone (**1**): to a stirred mixture of 304 mg of *C. schoenantus* essential oil dissolved in 3 ml of ethanol at 95° was added 1 mmol (111.5 mg) of semicarbazide hydrochloride dissolved in 2 ml of distilled water. 5 drops of triethylamine were added to a mixture after a minute of stirring. Then crystals appeared after 5 min of agitation but the stirring was maintained for another hour. The resulting crystals were filtered, washed until neutral, dried, weighed and then recrystallized in ethanol (Fig. 1). Piperitone substituted semicarbazone and thiosemicarbazones (**2**, **3**, **4**): To a stirring of the mixture of 304 mg of *C. schoenantus* essential oil dissolved in 3 ml of ethanol was added 1 mmol of semicarbazide or substituted (thio) semicarbazides dissolved in 3 ml of hydrochloric acid (1 N). After the appearance of crystals between one to three minutes, stirring was continued for one hour. The resulting crystals were filtered, washed until neutral, dried, weighed and recrystallized from ethanol (Fig. 2).

### 2.3 Bioassay tests

The Antitrypanosomal activity, toxicity test, and cytotoxicity assay were done according to Räz et al. [6], Sleet and Brendel [7] and Stevigny et al. [8], respectively.

## Figures and Tables

**Table 1:** Chemical composition of *Cymbopogon schoenantus* essential oil.

a**Compounds**	b**RI**	**%Area**
myrcene	991	0.18
δ-2-carene	1000	19.38
p-cymene	1025	0.13
limonene	1030	3.16
*Cis-*β-ocimene	1036	0.19
*trans*-β-ocimene	1047	0.13
*Cis-*menth-2-en-1-ol	1126	1.02
*trans*-menth-2-en-1-ol	1144	0.68
citronellal	1153	0.12
α-terpineol	1196	1.29
*trans*-piperitol	1210	0.37
eucarvone	1249	0.22
piperitone	1262	68.20
β-elemene	1390	0.21
β-caryophyllene	1421	0.40
elemol	1548	1.20
eranyle butyrate	1553	0.30
carayophyllene oxide	1584	0.20
γ-eudesmol	1632	0.20
α-cadinol	1656	0.53
**Total**		**98.11**

a Compounds listed in order of elution from HP-5-MS column.

b Retention indice (RI) on HP5-MS.

**Table 2:** *in vitro* antitrypanosomal, cytotoxicity and toxicity against *A. salina* Leach, and selectivity indices of hemi-synthesized compounds.

**Composés**		**1**	**2**	**3**	**4**
**Antitrypanosomal activity (IC**_**50**_**μM)**	*Tbb*	478.47±7.19^c^	74.58±4.44^b^	8.63±0.81^a^	10.90±2.52^a^
	activity	low	moderate	trypanocidal	trypanocidal
**Toxicity against*****A. salina*****Leach**	LC_50_ (μM)	373.20±6.60^c^	86.66±2.33^b^	85.52±2.28^b^	32.22±1.66^a^
	Activity	Not toxic	Not toxic	Not toxic	Toxic
					
**Cytotoxicity (IC**_**50**_**μM)**	WI38 (μM)	481.48±6.69^c^	143.38±4.89^b^	80.95±9.15^a^	134.09±5.45^b^
	Activity	Not cytotoxic	Not cytotoxic	Not cytotoxic	Not cytotoxic
	CHO (μM)	213.54±7.56^c^	21.16±1.37^a^	65.87±4.8^b^	65.35±4.02^b^
	Activity	Not toxic	Cytotoxic	Moderate	Moderate
					
^**α**^**Selectivity indices**	LC_50_/*tbb*	0.78	1.16	9.91	2.96
	WI38/ *tbb*	1.01	1.92	9.38	12.30
	CHO/*tbb*	0.45	0.28	7.63	6.00
	WI38/CHO	2.25	6.78	1.23	2.05

**1**: piperitone semicarbazone, **2**: piperitone thiosemicarbazone, **3**: piperitone 4-phenyl semicarbazone, **4**: piperitone 4-phenyl thiosemicarbazone. *Tbb*: *Trypanosoma brucei brucei*, ^α^Selectivity index (SI): IC_50_ (WI38)/IC_50_ (Tbb), IC_50_: sample concentration providing 50% death of cells or parasites, LC_50_: sample concentration providing 50% death of larvae, WI38: human normal fibroblast cells, CHO: Chinese Hamster Ovary cells; Data in the same line followed by different letters are statistically different by Student׳s *t*-test (*P*<0.05). Values are means±standard deviation of three separate experiments.
