# Supplementary material for: Structural and antitrypanosomal data of different carbasones of piperitone
Source: Data Brief. 2016 Nov 18;9:1039–43. doi: 10.1016/j.dib.2016.11.044 (PMC5123075; doi:10.1016/j.dib.2016.11.044)
Supplement: Supplementary file 2 — Supplementary material [file mmc2.docx]

**Supplementary data**

***Compound physical and spectrometric data***

**Piperitone semicarbazone (1) Yield:** 56%; m.p: 196-197°C; **IR** (NaCl, cm^-1^): 3635, 3530, 3440 ѵ(NH_2_); 3145 ѵ(NH); 1576 ѵ(C=N); 1671 ѵ(C=O); 1471, 1375, 1332 ѵ(C=C). **^13^C NMR** (CDCl_3_, 100 *MHz*) δ(ppm): 158; 146 ; 137 ; 128 ; 42 ; 39 ; 27 ; 22 ; 17 ; 16 ; **^1^H NMR** (CDCl_3_, 400 *MHz*), δ(ppm): 0.9 and 1.2 (d, 6H, -C(CH_3_)_2_); 1.6 (s, 3H, -CH_3_); 1.6 and 1.7 (m, 4H, 2CH_2_); 2,8 (m, 1H,N=CCH-); 2 (m, 1H, -CH(CH_3_)_2_); 5.6 (s, 1H, C=CH); 6.9 (s, 2H, CSNH_2_); 8.7 (s, 1H,=NNH−). **MS** (*m/z*): [M+H]^+^ 209.15; [M+H]^+^ found 209.04.

**Piperitone thiosemicarbazone (2) Yield:** 67%; m.p: 163-164°C; **IR** (NaCl, cm^-1^): 3587, 3523, 3446 ѵ(NH_2_); 3261 ѵ(NH); 1606 ѵ(C=N); 1495, 1476 ѵ(C=C); 831 ѵ(C=S). **^13^C NMR** (CDCl_3_, 100 *MHz*) δ(ppm): 182; 151; 123; 114 ; 43; 39; 24; 21; 20; 19 ; **^1^H NMR** (CDCl_3_, 400 *MHz*), δ(ppm): 1 and 1.2 (d, 6H, -C(CH_3_)_2_); 1.6 (s, 3H, -CH_3_); 1.8 and 2.1 (m, 4H, 2CH_2_); 2.2 (m, 1H, -CH(CH_3_)_2_); 2,3 (m, 1H, -CHC=N); 6.1 (s, 1H, C=CH); 7.2 (s, 2H, CSNH_2_); 8.9 (s, 1H,=NNH−). **MS** (*m/z*): [M+H]^+^ 225.13; [M+H]^+^ found 225.04.

**4-phenyl piperitone semicarbazone (3) Yield:** 71%; m.p: 165-166°C; **IR** (NaCl, cm^-1^): 3607, 3529, 3442 ѵ(NH2); 3198 ѵ(NH); 1685 ѵ(C=O); 1602 ѵ(C=N); 1499, 1447 ѵ(C=C). **^13^C NMR** (CDCl_3_, 100 *MHz*) δ(ppm): 154; 145; 133 ; 128 ; 126 ; 118 ; 39; 36 ; 28 ; 25 ; 19 ; 18 ; 17; **^1^H NMR** (CDCl_3_, 400MHz), δ(ppm): 0.8 and 1(d, 6H, -C(CH_3_)_2_); 1.3 (s, 3H, -CH_3_); 1.7 and 2 (m, 4H, 2CH_2_); 2,7 (m, 1H, N=CCH); 2.4 (m, 1H, -CH(CH_3_)_2_); 5.6 (s, 1H, C=CH); H aromatic : 7.2, 7.3, 7.5 ; 8.3 (s, 2H, CSNH_2_); 9.1; 8.9 (s, 1H,=NNH−). **MS** (m/z): [MH]^+^ 285.18; [MH]^+^ found 285.06.

**4-phenyl piperitone thiosemicarbazone (4) Yield:** 87%; m.p: 120-121°C; **IR** (NaCl, cm^-1^): 3522, 3445 ѵ(NH2); 3323 ѵ(NH); 1591 ѵ(C=N); 1539, 1487 ѵ(C=C); 853 ѵ(C=S). **^13^C NMR** (CDCl_3_, 100 *MHz*) δ(ppm):; **^1^H NMR** (CDCl_3_, 400 *MHz*), δ(ppm): 0.8 and 1.1 (d, 6H, -C(CH_3_)_2_); 1.5 (s, 3H, -CH_3_); 1.8 and 2 (m, 4H, 2CH_2_); 2,6 (m, 1H, N=CCH-); 2.4 (m, 1H, -CH(CH_3_)_2_); 5.9, 6.3 (s, 1H, CH=C); H aromatics: 7.1, 7.3 7.7 ; 8.7, 8.8 (s, 2H, CSNH_2_); 9.3 (s, 1H,=NNH−). **MS** (m/z): [MH]^+^ 301.16; [MH]^+^ found 301.03.
